# Supplementary material for: Microplastic burden in marine benthic invertebrates depends on species traits and feeding ecology within biogeographical provinces
Source: Nat Commun. 2023 Dec 4;14:8023. doi: 10.1038/s41467-023-43788-w (PMC10696022; doi:10.1038/s41467-023-43788-w)
Supplement: Supplementary file 1 — Supplementary Information [file 41467_2023_43788_MOESM1_ESM.pdf]

Supplementary Materials

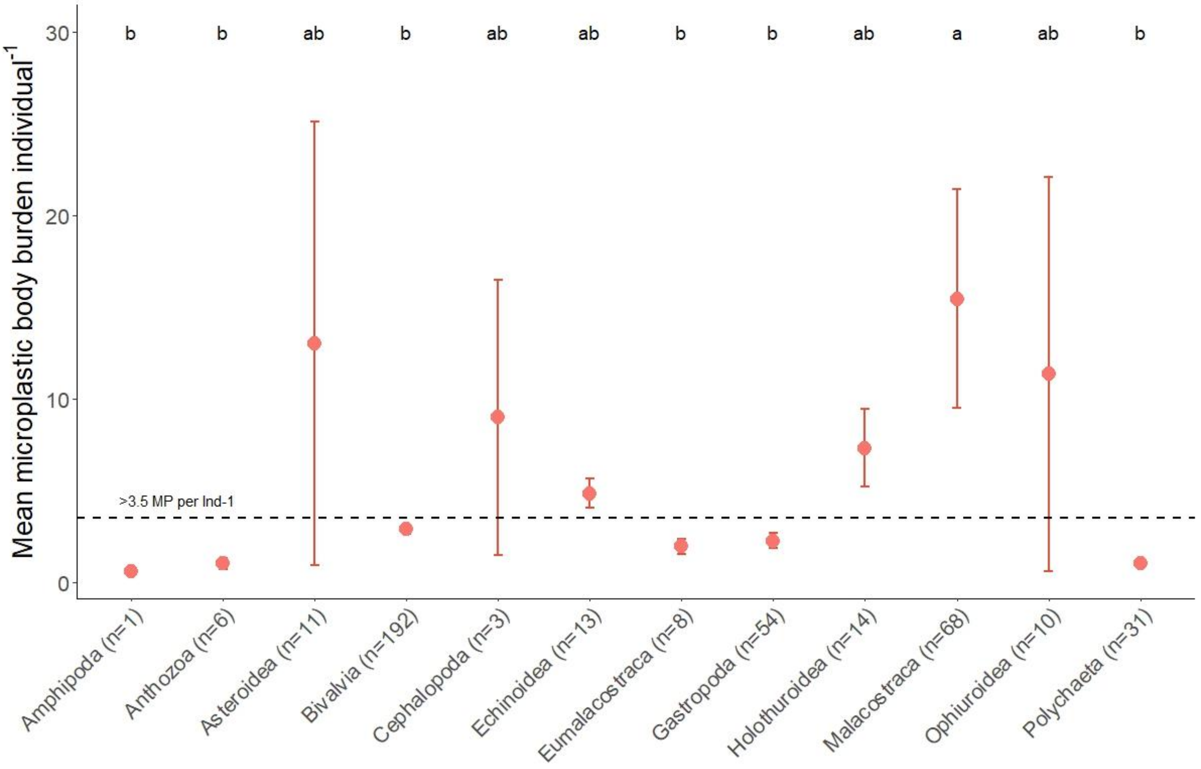

Figure S1: The mean (±s.e.) microplastic body burden individual^{-1} in marine benthic invertebrate fauna represented by taxonomic class. Statistically significant groupings are indicated by letter.

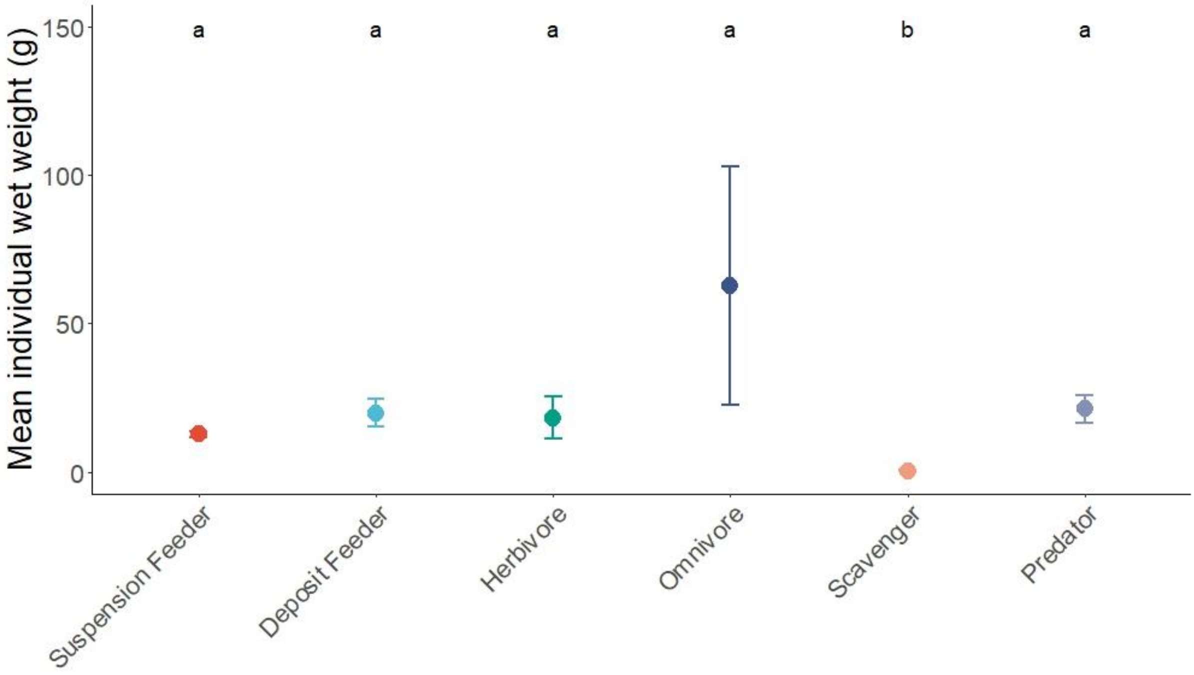

Figure S2: Observed mean wet weight (± s.e.) of organisms from each feeding mode. Distinct groupings are indicated (lower case lettering) identified from examination of model coefficients following analysis with a linear model incorporating generalized least-squares (GLS) estimation to allow the residual spread to vary with feeding group.

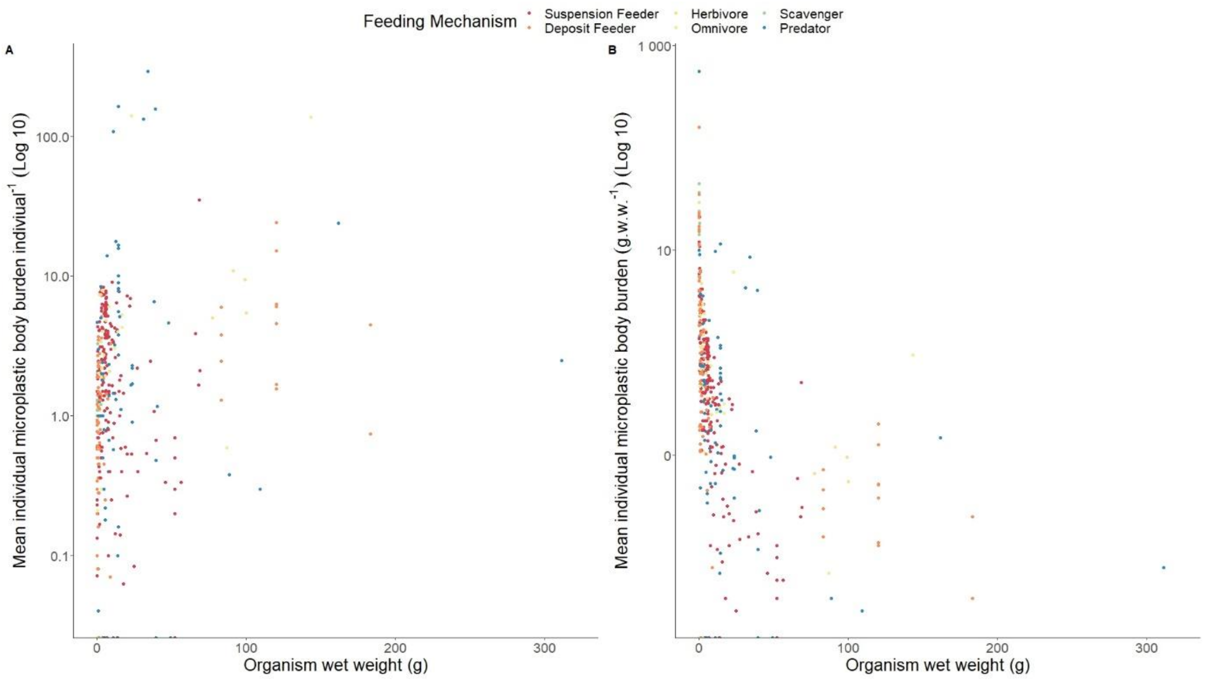

Figure S3: Reported organismal weight plotted against (A) mean individual microplastic body individual^{-1} and (B) standardized by body size using mean individual microplastic body burden gram wet weight^{-1} for contrasting feeding modes of marine benthic invertebrates. We found no evidence that individual microplastic body burden corresponded to biomass (wet weight ind.$^{-1}$), irrespective of whether the microplastic burden was reported per individual (r (410) = 0.09, p = 0.059, n = 412) or per unit biomass (g wet weight^{-1}; (r (410) = -0.05, p = 0.337, n = 412).

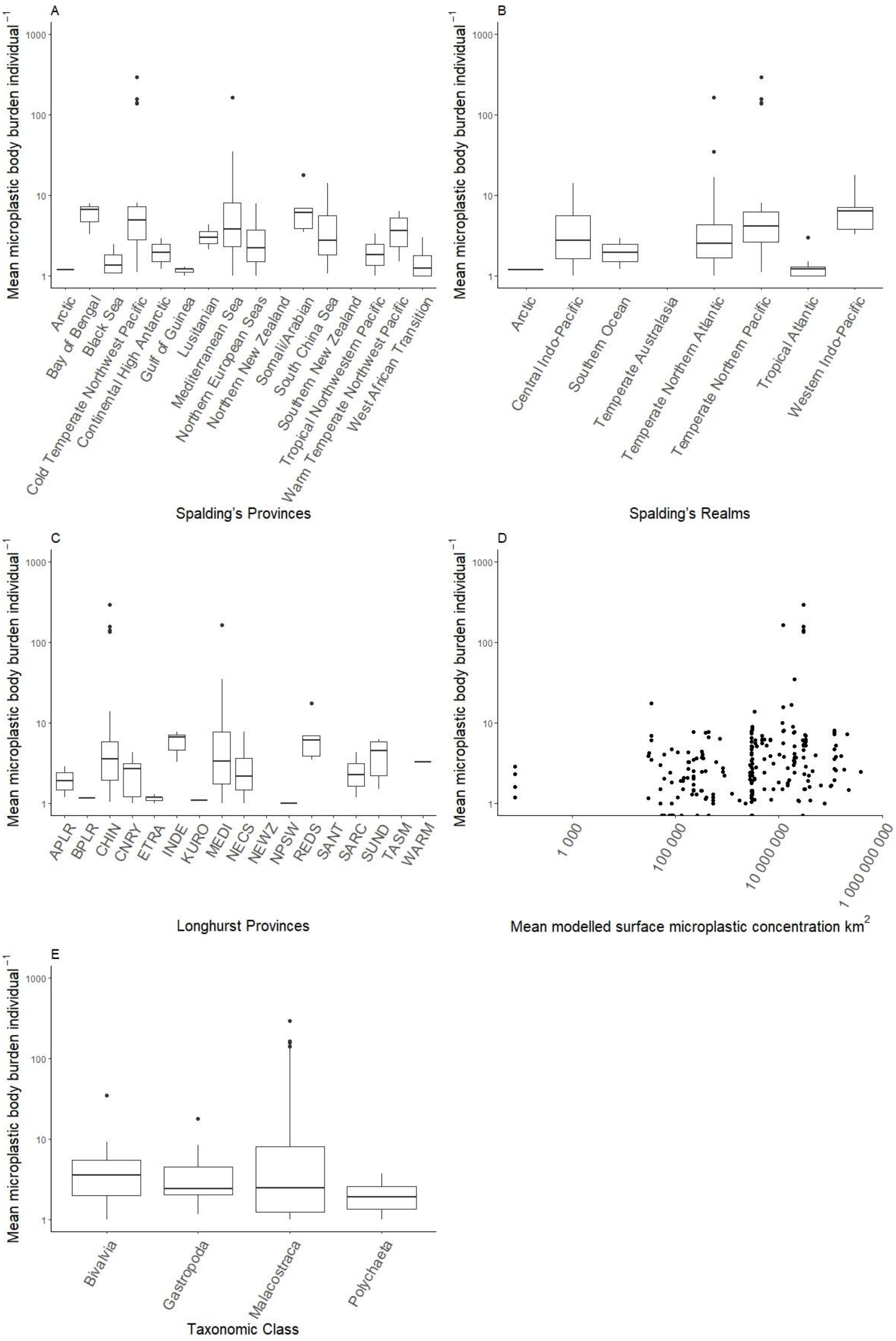

Figure S4: Mean individual microplastic body burden individual-1 for: (A) Spalding's Provinces (F(15,330) = 2.427, p = 0.0023), (B) Spalding's Realms (F(7,338) = 2.522, p = 0.015), (C) Longhurst Province (F(16,329) = 0.369, p = 0.988), (D) mean microplastic contamination per km^2, as predicted by the Van Sebille et al. model, within a 250 km radius of the sampled organism record (r(344) = 0.154, p = 0.878) (E) taxonomic class

(F(3,342) = 6.212, p < 0.001). From these plots Spalding's provinces and taxonomic class were taken forward as explanatory variables in our model. Longhurst province codes in panel C are: INDE: Coastal - E. India Coastal Province; MEDI: Westerlies - Mediterranean Sea, Black Sea Province; APLR: Polar - Austral Polar Province; CHIN: Coastal - China Sea Coastal Province; NEWZ: Coastal - New Zealand Coastal Province; TASM: Westerlies - Tasman Sea Province; SANT: Westerlies - Subantarctic Province; NECS: Coastal - NE Atlantic Shelves Province; SUND: Coastal - Sunda-Arafura Shelves Province; KURO: Westerlies - Kuroshio Current Province; NPSW: Westerlies - N. Pacific Subtropical Gyre Province (West); WARM: Trades - W. Pacific Warm Pool Province; ETRA: Trades - Eastern Tropical Atlantic Province; SARC: Polar - Atlantic Subarctic Province; BPLR: Polar - Boreal Polar Province (POLR); REDS: Coastal - Red Sea, Persian Gulf Province; CNRY: Coastal - Canary Coastal Province (EACB). Boxplots show the inter-quartile range (box) with median line. All continuous scales are presented as Log10 to improve the data clarity.

Supplementary Note 1 - Search Terms

"((TS= ((microplastic* OR micro-plastic*) OR (plastic*) AND (benth*))
NOT TS= (plant* OR terrestrial* OR soil* OR river* OR fluvial OR freshwater OR adsorp* OR sorp* OR zebrafish OR mice OR transcipt* OR cell culture OR waste management OR molecular OR degradation OR biodegradation OR bird* OR salt)
NOT TI= (policy OR beach OR sea surface OR air OR atmospheric OR drinking water OR lake OR plasticity OR antibiotic OR antimicrob* Or tap water OR bottled water OR human OR floating)
NOT WC= (Physics, Applied OR Physics, Atomic, Molecular & Chemical OR Physics, Fluids & Plasmas OR Physics, Mathematical OR Physics, Multidisciplinary OR Physics, Nuclear OR Physics, Particles & Fields OR Engineering, Aerospace OR Engineering, Biomedical OR Engineering, Chemical OR Engineering, Civil OR Engineering, Electrical & Electronic OR Engineering, Environmental OR Engineering, Geological OR Engineering, Industrial OR Engineering, Manufacturing OR Engineering, Marine OR Engineering, Mechanical OR Engineering, Multidisciplinary OR Engineering, Ocean OR Engineering, Petroleum OR Materials Science, Biomaterials OR Materials Science, Ceramics OR Materials Science, Characterization & Testing OR Materials Science, Coatings & Films OR Materials Science, Composites OR Materials Science, Multidisciplinary OR Materials Science, Paper & Wood OR Materials Science, Textiles OR Genetics & Heredity OR Geochemistry & Geophysics OR Geology OR Geosciences, Multidisciplinary OR Social Issues OR Social Sciences, Biomedical OR Social Sciences, Interdisciplinary OR Social Sciences, Mathematical Methods OR Sociology OR Oceanography OR Law)))"
Timespan=2000-2020. Indexes=SCI-EXPANDED, A&HCI, CPCI-S, ESCI.

Search Field Tag Descriptions

1. TS = Searches for topic terms in the Title, Abstract and Keyword fields within a record.
2. NOT = Excludes records containing certain words from your search.
3. OR = Finds records containing any of the terms separated by the operator
4. * = Wildcard representation for unknown characters used to capture all derivatives of words including plurals. (e.g. transciptomics, transcriptome etc.)
5. TI = Searches the Title field within a record.
6. WC = A Web of Science category; used here to filter out non-subject specific references (e.g. plasticity is referred to in genetics and material science)
7. Timespan = The year the record was published.
8. Indexes = Web of Science core collection database comprises a number of indexes; Science Citation Index Expanded (SCI-EXPANDED), Arts & Humanities Citation Index (A&HCI), Conference Proceedings Citation Index- Science (CPCI-S), and Emerging Sources Citation Index (ESCI) in this case.

Supplementary Table 1: Eligibility criteria for article screening for the study question.

Question elements	Eligibility criteria	
	**Included**	**Excluded**
**Populations**	Marine or marine related, benthic dwelling, invertebrate species (including estuarine and salt, marsh species)	Non-marine species and all marine fishes
		Sediment contamination with microplastics
	Studies with individuals collected from fish markets if collection grounds were reported	
**Intervention**	Studies that looked at microplastic uptake in environmentally collected organisms	Laboratory studies
		Specifically nano- or macro-plastic studies
**Comparators**	Studies that were published from 2000 - 2020	Pre 2000 studies due to a lack of QA/QC procedures (see below in study design)
	NA	NA
**Outcomes**	Microplastic ingestion in particles per individual or particles per gram wet weight of tissue.	Studies which measured frequency of occurrence or presence/absence only.
		Studies which only reported particles per individual *of those that had ingested plastic* - if the total number of individuals collected was reported the data were corrected, if not the papers were excluded.
**Study design**	Must have atmospheric and procedural contamination controls	Reviews
	Must use some form of spectroscopic confirmation of plastics (e.g. FTIR or Raman spectroscopy)	Focus on one shape of plastics (e.g. paint particles only or fibres only)
		Studies that due to methodological constraints chose not to count certain particle types

		Hot needle test as a plastic confirmation technique
		Studies that focussed on method development (often digestion techniques or recovery rates)
		Studies that only used chemical presence to infer plastic uptake (e.g. phthalic acid esters (PAEs))
		Observations from platforms such as ROVs or drone footage showing adherence to plastics
		Studies where species were pooled for digestion and reported as particle per gram of tissue

Supplementary Table 2: Microplastic size shape and polymer data collected from the primary literature. These particles were recovered from benthic macroinvertebrates and most studies reported percentage contribution to the total number of particles recovered. Different numbers of studies reported different characteristics and the number of studies data were collected from are represented in the table.

Microplastic Characteristic	Descriptor	Average % Contribution	±s.e.
Polymer (388 Studies)	Cellulose	36.6	0.80
	Polyethylene Terephthalate	15.6	1.06
	Polyethylene	12.1	0.63
	Polyamide	7.5	0.65
	Polypropylene	7.3	0.31
	Nylon	4.6	0.21
	Polyester	4.2	0.05
	Other	3.5	0.08
	Acrylic	3.5	0.56
	Ethylene Vinyl Acetate	1.5	1.70
	Polystyrene	1.3	0.13
	Polyvinyl Chloride	1.3	0.81
	Acrylonitrile Butadiene Styrene	0.4	0.35
	Styrene-Butadiene Rubber	0.3	0.18
	Polyurethane	0.2	0.38
	Polyvinyl Acetate	0.2	0.33
Colour (237 studies)	Blue	27.7	1.24
	Clear	19.6	0.99
	Black/Grey	15.5	1.03
	White	14	0.88
	Red	11.1	0.32
	Green	3.8	1.87
	Other	3.3	0.19
	Orange	2.9	0.16
	Yellow	2	0.40
Shape (397 Studies)	Fibres	75.0	1.46
	Fragments	16.8	1.16
	Spheres	3.6	0.71
	Foams	0.1	0.02
	Films	2.6	0.38
	Pellets	1.9	0.15
	Other	0.01	0.01
