# Supplementary material for: Microplastic burden in marine benthic invertebrates depends on species traits and feeding ecology within biogeographical provinces
Source: Nat Commun. 2023 Dec 4;14:8023. doi: 10.1038/s41467-023-43788-w (PMC10696022; doi:10.1038/s41467-023-43788-w)
Supplement: Supplementary file 3 — Reporting Summary [file 41467_2023_43788_MOESM3_ESM.pdf]

Corresponding author(s): Dr Adam Porter

Last updated by author(s): Nov 9, 2023

Reporting Summary

Nature Portfolio wishes to improve the reproducibility of the work that we publish. This form provides structure for consistency and transparency in reporting. For further information on Nature Portfolio policies, see our Editorial Policies and the Editorial Policy Checklist.

Statistics

For all statistical analyses, confirm that the following items are present in the figure legend, table legend, main text, or Methods section.

n/a	Confirmed	
☐	☒	The exact sample size (*n*) for each experimental group/condition, given as a discrete number and unit of measurement
☐	☒	A statement on whether measurements were taken from distinct samples or whether the same sample was measured repeatedly
☐	☒	The statistical test(s) used AND whether they are one- or two-sided<br>*Only common tests should be described solely by name; describe more complex techniques in the Methods section.*
☐	☒	A description of all covariates tested
☐	☒	A description of any assumptions or corrections, such as tests of normality and adjustment for multiple comparisons
☐	☒	A full description of the statistical parameters including central tendency (e.g. means) or other basic estimates (e.g. regression coefficient) AND variation (e.g. standard deviation) or associated estimates of uncertainty (e.g. confidence intervals)
☐	☒	For null hypothesis testing, the test statistic (e.g. *F*, *t*, *r*) with confidence intervals, effect sizes, degrees of freedom and *P* value noted<br>*Give P values as exact values whenever suitable.*
☒	☐	For Bayesian analysis, information on the choice of priors and Markov chain Monte Carlo settings
☒	☐	For hierarchical and complex designs, identification of the appropriate level for tests and full reporting of outcomes
☐	☒	Estimates of effect sizes (e.g. Cohen's *d*, Pearson's *r*), indicating how they were calculated

Our web collection on statistics for biologists contains articles on many of the points above.

Software and code

Policy information about availability of computer code

Data collection

Data were curated using the following boolean search string using Web of Science (https://www.webofscience.com/): "((TS= ((microplastic* OR micro-plastic*) OR (plastic*) AND (benth*))
NOT TS= (plant* OR terrestrial* OR soil* OR river* OR fluvial OR freshwater OR adsorp* OR sorp* OR zebrafish OR mice OR transcipt* OR cell culture OR waste management OR molecular OR degradation OR biodegradation OR bird* OR salt)
NOT TI= (policy OR beach OR sea surface OR air OR atmospheric OR drinking water OR lake OR plasticity OR antibiotic OR antimicrob* Or tap water OR bottled water OR human OR floating)
NOT WC= (Physics, Applied OR Physics, Atomic, Molecular & Chemical OR Physics, Fluids & Plasmas OR Physics, Mathematical OR Physics, Multidisciplinary OR Physics, Nuclear OR Physics, Particles & Fields OR Engineering, Aerospace OR Engineering, Biomedical OR Engineering, Chemical OR Engineering, Civil OR Engineering, Electrical & Electronic OR Engineering, Environmental OR Engineering, Geological OR Engineering, Industrial OR Engineering, Manufacturing OR Engineering, Marine OR Engineering, Mechanical OR Engineering, Multidisciplinary OR Engineering, Ocean OR Engineering, Petroleum OR Materials Science, Biomaterials OR Materials Science, Ceramics OR Materials Science, Characterization & Testing OR Materials Science, Coatings & Films OR Materials Science, Composites OR Materials Science, Multidisciplinary OR Materials Science, Paper & Wood OR Materials Science, Textiles OR Genetics & Heredity OR Geochemistry & Geophysics OR Geology OR Geosciences, Multidisciplinary OR Social Issues OR Social Sciences, Biomedical OR Social Sciences, Interdisciplinary OR Social Sciences, Mathematical Methods OR Sociology OR Oceanography OR Law)))"
Timespan=2000-2020. Indexes=SCI-EXPANDED, A&HCI, CPCI-S, ESCI.

The data used from the Van Sebille Model (2015) (DOI: 10.1088/1748-9326/10/12/124006) were downloaded from: https://figshare.com/collections/data_of_Van_Sebille_et_al_2015_ERL_paper/5764184.

The Spaldings ecoregion shapefiles were downloaded from: https://www.worldwildlife.org/publications/marine-ecoregions-of-the-world-a-

bioregionalization-of-coastal-and-shelf-areas

Longhurst province shapefiles were downloaded from: https://www.marineregions.org/gazetteer.php?p=details&id=22538

WebPlotDigitizer (https://automeris.io/WebPlotDigitizer/; version 4.6) was used to gather data from published figures.

Data analysis

ArcGIS Desktop v. 10.8 were used to build maps.

Data analyses were primarily undertaken in R (R Core Team (2023; version 2023.03.0 Build 386) using the following packages: nmle and agricolae.

PCAs were performed in PRIMER (version 6.1.13)

For manuscripts utilizing custom algorithms or software that are central to the research but not yet described in published literature, software must be made available to editors and reviewers. We strongly encourage code deposition in a community repository (e.g. GitHub). See the Nature Portfolio guidelines for submitting code & software for further information.

Data

Policy information about availability of data

All manuscripts must include a data availability statement. This statement should provide the following information, where applicable:

- Accession codes, unique identifiers, or web links for publicly available datasets
- A description of any restrictions on data availability
- For clinical datasets or third party data, please ensure that the statement adheres to our policy

The research data supporting this publication are openly available from Harvard Dataverse at: https://doi.org/10.7910/DVN/E57LOA. The data for the Van Sebille 2015 model can be found at: https://figshare.com/collections/data_of_Van_Sebille_et_al_2015_ERL_paper/5764184. Ocean boundaries (Spalding's Provinces) used in Figure 1 are freely available at: https://www.worldwildlife.org/publications/marine-ecoregions-of-the-world-a-bioregionalization-of-coastal-and-shelf-areas. Longhurst provinces used as a geographical variable in the initial analysis are freely available from: https://www.marineregions.org/gazetteer.php?p=details&id=22538. The world country shapefiles used in Figure 1 are available from ESRI at: https://hub.arcgis.com/datasets/esri::world-countries-generalized/about and available for use under the ESRI Master License Agreement. Taxonomy for all species was verified and curated using the World Register of Marine Species match taxa function available at: https://www.marinespecies.org/aphia.php?p=match. Biological trait categories were modified using those provided by the Marine Life Information Network (MarLIN) Biological Traits Information Catalogue (BIOTIC) available at: https://www.marlin.ac.uk/biotic/resources.php. Latitudes and Longitudes when not specifically mentioned in the individual study were approximated using Google Maps.

Research involving human participants, their data, or biological material

Policy information about studies with human participants or human data. See also policy information about sex, gender (identity/presentation), and sexual orientation and race, ethnicity and racism.

Reporting on sex and gender	not applicable
Reporting on race, ethnicity, or other socially relevant groupings	not applicable
Population characteristics	not applicable
Recruitment	not applicable
Ethics oversight	not applicable

Note that full information on the approval of the study protocol must also be provided in the manuscript.

Field-specific reporting

Please select the one below that is the best fit for your research. If you are not sure, read the appropriate sections before making your selection.

☐ Life sciences ☐ Behavioural & social sciences ☒ Ecological, evolutionary & environmental sciences

For a reference copy of the document with all sections, see nature.com/documents/nr-reporting-summary-flat.pdf

Ecological, evolutionary & environmental sciences study design

All studies must disclose on these points even when the disclosure is negative.

Study description	A global meta-analysis looking at the relationship between the amount of microplastic in benthic marine invertebrates and biological traits that infer a greater risk of uptake of microplastics.

Research sample	These data are curated from 55 primary research articles, collected using a systematic sampling strategy from the available literature using the Web of Science (https://www.webofscience.com/wos/woscc/basic-search) search engine (see below). The inital search returned 1851 research articles which were filtered by title relevance, followed by abstract relevance. This screening returned 96 studies to be screened using our inclusion/exclusion criteria and the final 55 had data extracted from them. The search spanned the years 2000-2020 as this represents a period of time when relevant quality control measures have been used consistently in the microplastic literature. This produced a dataset comprising 412 individual species records. We investigated what geographic, taxonomic, or biological factors conferred an elevated body burden of microplastic.
Sampling strategy	Studies included in the study were collected using the following Boolean Search string: "((TS= ((microplastic* OR micro-plastic*) OR (plastic*) AND (benth*))<br>NOT TS= (plant* OR terrestrial* OR soil* OR river* OR fluvial OR freshwater OR adsorp* OR sorp* OR zebrafish OR mice OR transcipt* OR cell culture OR waste management OR molecular OR degradation OR biodegradation OR bird* OR salt)<br>NOT TI= (policy OR beach OR sea surface OR air OR atmospheric OR drinking water OR lake OR plasticity OR antibiotic OR antimicrob* Or tap water OR bottled water OR human OR floating)<br>NOT WC= (Physics, Applied OR Physics, Atomic, Molecular & Chemical OR Physics, Fluids & Plasmas OR Physics, Mathematical OR Physics, Multidisciplinary OR Physics, Nuclear OR Physics, Particles & Fields OR Engineering, Aerospace OR Engineering, Biomedical OR Engineering, Chemical OR Engineering, Civil OR Engineering, Electrical & Electronic OR Engineering, Environmental OR Engineering, Geological OR Engineering, Industrial OR Engineering, Manufacturing OR Engineering, Marine OR Engineering, Mechanical OR Engineering, Multidisciplinary OR Engineering, Ocean OR Engineering, Petroleum OR Materials Science, Biomaterials OR Materials Science, Ceramics OR Materials Science, Characterization & Testing OR Materials Science, Coatings & Films OR Materials Science, Composites OR Materials Science, Multidisciplinary OR Materials Science, Paper & Wood OR Materials Science, Textiles OR Genetics & Heredity OR Geochemistry & Geophysics OR Geology OR Geosciences, Multidisciplinary OR Social Issues OR Social Sciences, Biomedical OR Social Sciences, Interdisciplinary OR Social Sciences, Mathematical Methods OR Sociology OR Oceanography OR Law)))"<br>Timespan=2000-2020. Indexes=SCI-EXPANDED, A&HCI, CPCI-S, ESCI.<br><br>They were then screened using the PICO (Problem, Intervention, Comparison, Outcome) framework. The specific inclusion and exclusion criteria are reported in detail in the manuscript and supplementary information.
Data collection	Data were curated from the available literature using the Clarivate Web of Science (https://www.webofknowledge.com/) to source peer-reviewed articles that contained measurements of microplastic body burden using specific search terms
Timing and spatial scale	Studies included were published between 2000 and 2020.
Data exclusions	To be included in our quantitative synthesis each study had to meet the following criteria: (1) an empirical study focussing on marine associated and benthic dwelling invertebrate species; (2) the focus was on microplastic exposure; (3) the organisms must be field collected organisms rather than laboratory studies; (4) the study must report particles per individual or particles per gram of wet weight of tissue; (5) evidence of quality assurances such as contamination control and spectroscopic confirmation of plastic presence were required.
Reproducibility	All the data have been made publicly available.
Randomization	Not applicable - all traits were assigned to all species based on peer reviewed research and expert opinion and not randomly. allocated.
Blinding	Blinding was not necessary as strict inclusion/exclusion criteria were set ensuring that objectivity was maintained in article selection.

Did the study involve field work? ☐ Yes ☒ No

Reporting for specific materials, systems and methods

We require information from authors about some types of materials, experimental systems and methods used in many studies. Here, indicate whether each material, system or method listed is relevant to your study. If you are not sure if a list item applies to your research, read the appropriate section before selecting a response.

Materials & experimental systems

n/a	Involved in the study	
☒	☐	Antibodies
☒	☐	Eukaryotic cell lines
☒	☐	Palaeontology and archaeology
☒	☐	Animals and other organisms
☒	☐	Clinical data
☒	☐	Dual use research of concern
☒	☐	Plants

Methods

n/a	Involved in the study	
☒	☐	ChIP-seq
☒	☐	Flow cytometry
☒	☐	MRI-based neuroimaging

Plants

Seed stocks	*Report on the source of all seed stocks or other plant material used. If applicable, state the seed stock centre and catalogue number. If plant specimens were collected from the field, describe the collection location, date and sampling procedures.*
Novel plant genotypes	*Describe the methods by which all novel plant genotypes were produced. This includes those generated by transgenic approaches, gene editing, chemical/radiation-based mutagenesis and hybridization. For transgenic lines, describe the transformation method, the number of independent lines analyzed and the generation upon which experiments were performed. For gene-edited lines, describe the editor used, the endogenous sequence targeted for editing, the targeting guide RNA sequence (if applicable) and how the editor was applied.*
Authentication	*Describe any authentication procedures for each seed stock used or novel genotype generated. Describe any experiments used to assess the effect of a mutation and, where applicable, how potential secondary effects (e.g. second site T-DNA insertions, mosiacism, off-target gene editing) were examined.*
